# Antimicrobial susceptibility of priority bacterial pathogens from laboratory samples in Timor-Leste: a retrospective analysis of database (2020–2025)

**DOI:** 10.1016/j.lansea.2026.100847

**Published:** 2026-08-20

**Authors:** Tessa Oakley, Carolina da Costa Maia, Celita Osorio do Rosario, Messias Soares, Joana Correia Belo, Virginia da Conceição, Névio Sarmento, Lucia Toto, Elfiana Amaral, Domingas da Costa Campos, Raikos Allan, Karen Champlin, Endang Soares da Silva, Ari Jayanti Pereira Tilman, Helio Guterres, Lucsendar Alves, Anthony DK. Draper, Benjamin F.R. Dickson, Ian Marr, Jennifer Yan, Heidi Smith-Vaughan, Joshua R. Francis

**Affiliations:** a Menzies School of Health Research, Charles Darwin University, Dili, Timor-Leste; b Laboratório da Saúde do INSP-TL, Dili, Timor-Leste; c Instituto Nacional de Saúde Pública de Timor-Leste, Dili, Timor-Leste; d Maluk Timor, Dili, Timor-Leste; e Partnership for Human Development, Dili, Timor-Leste; f Hospitál Nacionál Guido Valadares, Dili, Timor-Leste; g National Centre for Epidemiology and Population Health, Australian National University, Canberra, Australia; h Centre for Disease Control, Northern Territory Government Department of Health, Casuarina, Australia; i The Canberra Hospital, ACT, Australia; j Royal Darwin Hospital, NT, Australia

**Keywords:** Antimicrobial resistance, Antimicrobial susceptibility, Enterobacterales, Extended-spectrum β-lactamase, ESBL, Carbapenem resistance, AMR surveillance, Timor-Leste, Global antimicrobial resistance surveillance system (GLASS)

## Abstract

**Background:**

Antimicrobial resistance (AMR) is a major global health threat, with the highest burden in low-income and middle-income countries where diagnostic and surveillance capacity are often limited. Over the past decade, Timor-Leste has made substantial progress in strengthening microbial diagnostics and AMR surveillance capacity. We aimed to describe antimicrobial susceptibility patterns and temporal trends among priority bacterial pathogens in Timor-Leste between 2020 and 2025.

**Methods:**

We retrospectively analysed antimicrobial susceptibility patterns for WHO priority pathogens isolated from clinical samples processed at the Laboratory of Health, Instituto Nacional de Saúde Pública de Timor-Leste (INSP-TL), between June 2020 and January 2025. WHO Global Antimicrobial Resistance and Use Surveillance System (GLASS) priority pathogens were identified using Matrix-Assisted Laser Desorption/Ionization Time-of-Flight mass spectrometry (MALDI-ToF) with automated antimicrobial susceptibility testing (AST) and interpreted using European Committee on AST (EUCAST) breakpoints. Duplicates within 12 months were excluded. Susceptibility proportions, temporal trends, and associations of AMR with sex, age, specimen type, and care were analysed.

**Findings:**

Of 4169 isolates, 2170 (52%) were from male and 1943 (47%) from female patients. Among Enterobacterales (1146/4169, 27%) ceftriaxone susceptibility of 65% (410/631) was observed in *Escherichia coli* and 25% (135/541) in *Klebsiella pneumoniae*. Carbapenem resistance was detected in 235 isolates, including 61 multidrug-resistant *Acinetobacter baumannii* complex. Meropenem susceptibility in *E. coli* decreased from 100% (33/33) in 2020 to 87% (155/178) in 2024 (p = 0.0002). Inpatient isolates of *E. coli* and *K. pneumoniae* showed lower ceftriaxone susceptibility than outpatient isolates. *Staphylococcus aureus* remained predominantly methicillin-susceptible (83%, 712/858).

**Interpretation:**

AMR burden in Timor-Leste is characterised by third-generation cephalosporin resistance, emerging carbapenem-resistant Enterobacterales, and multidrug-resistant *A. baumannii*. Sustained investment in infection prevention, antimicrobial stewardship, and laboratory systems is essential to mitigate further resistance emergence and guide treatment.

**Funding:**

This work was supported by a Fleming Fund Country Grant (UK Aid).

**Research in context**

**Evidence before this study**

Antimicrobial resistance (AMR) is a major global health challenge, with the highest burden falling on low- and middle-income countries where diagnostic capacity for surveillance is limited. We searched PubMed and Google Scholar for articles published up to February 2026, using combinations of the terms (“antimicrobial resistance” OR “antibiotic susceptibility” OR “antimicrobial susceptibility”) AND (“Timor-Leste” OR “East Timor” OR “Southeast Asia” OR “Pacific Islands”), without language restriction. Evidence from Southeast Asia and the Pacific highlights widespread extended-spectrum β-lactamase (ESBL) production among Enterobacterales and increasing carbapenem resistance, particularly in *Acinetobacter baumannii* and *Pseudomonas aeruginosa*. Data from Timor-Leste are limited, comprising small outbreak investigations and isolated laboratory summaries, with no longitudinal analysis of national AMR patterns and no comprehensive analysis of routine antimicrobial susceptibility testing (AST) data.

**Added value of this study**

This study retrospectively analysed antimicrobial susceptibility patterns for WHO priority pathogens isolated from clinical samples processed at the Laboratory of Health in Timor Leste, between June 2020 and January 2025. From 4169 isolates, we found a high burden of resistance among priority pathogens. Susceptibility to third-generation cephalosporins was low in *Escherichia coli* and *Klebsiella pneumoniae* (ceftriaxone 65% and 25%, respectively), indicative of potential widespread ESBL production, and carbapenem resistance was identified in 235 isolates. We documented the emergence of carbapenem-resistant *E. coli* with meropenem susceptibility declining from 100% in 2020 to 87% in 2024. Resistance was consistently higher among inpatient and intensive care isolates than outpatient isolates, and we identified statistically significant associations between reduced susceptibility and patient sex, age group, specimen type, and care setting, indicating a substantial hospital-associated component to the resistance burden. These findings establish a national baseline of antimicrobial susceptibility against which future trends can be assessed.

**Implications of all the available evidence**

Our findings indicate a substantial and growing AMR burden in Timor-Leste, characterised by widespread resistance to third-generation cephalosporins and emerging carbapenem resistance among Enterobacterales, alongside endemic multidrug-resistant *A. baumannii*. These patterns present major challenges in a setting with limited therapeutic options. Strengthened diagnostic systems have improved the detection of AMR. Sustained investment in laboratory capacity, infection prevention and control, and antimicrobial stewardship will be essential to prevent further resistance emergence. Future research incorporating clinical data, molecular characterisation, and broader multi-site sampling will be critical to guide treatment guidelines and national AMR policy.

## Introduction

Antimicrobial resistance (AMR) is an emerging threat to public health worldwide^1^ with a disproportionately high burden in low-and middle-income countries (LMIC).^1^, ^2^, ^3^ To address the growing burden, it is critical to establish and strengthen the diagnostic capacity to inform clinical management of infections and support ongoing surveillance. Currently, many LMIC lack the facilities and expertise to effectively identify and monitor the extent of AMR in their countries.^4^^,^^5^ Expanding diagnostic microbiology capacity and access to rapid, accurate diagnostic testing would substantially strengthen global AMR surveillance and support diagnostic stewardship by enabling laboratory guided clinical decisions and appropriate antimicrobial use.^6^ Improving AMR surveillance, is critical for antimicrobial stewardship initiatives and hospital infection prevention and control programs, and potentially reduce rates of AMR.

Timor-Leste has a population of almost 1.3 million, 70% of whom live in rural or remote mountainous areas.^7^ The Healthcare system rebuilding of the country has been motivated by an early and sustained commitment by the Government. Around 75% of total health services are delivered by the public sector, encompassing 193 health posts, 66 community health sectors, 5 municipality hospitals and one national hospital across the countries 13 districts.^8^

Prior to 2017, diagnostic capacity for antimicrobial resistance (AMR) surveillance in Timor-Leste was limited. Only minimal testing was performed for blood culture, urine and wound specimens, resulting in limited data on AMR patterns.^9^ A 2017 study investigating AMR in urine and skin isolates highlighted the absence of in-country diagnostic microbiology facilities with isolates collected for the study requiring shipment to Australia for confirmatory bacterial identification (ID) and antimicrobial susceptibility testing (AST).^10^ Before the introduction of automated diagnostic platforms, laboratory testing in Timor-Leste relied on manual biochemical identification methods (Analytical Profile Index (API), bioMérieux, France) and disc diffusion AST using antibiotic discs from multiple suppliers, some of uncertain quality. Quality control procedures for media and discs were performed inconsistently, and when control failures occurred, follow-up investigations were rarely conducted.

Since 2020, Timor-Leste has experienced an increase in AMR diagnostic capacity at the referral laboratory in the capital city of Dili.^11^^,^^12^ Sample submissions to the laboratory rose substantially, from an average of 330 samples per month in 2021 to 810 per month in 2024. Installation of a Laboratory Information Management System (SchuyLab CGM LIMS) and online results portal have allowed standardisation of results reporting, and easier data analysis for generation of antibiograms, which are integral to functional AST in microbiology.^13^

In 2020, the Laboratory of Health of the National Institute of Public Health, Timor-Leste (LoH, INSP-TL; formerly the National Health Laboratory) contributed data to the World Health Organization’s GLASS for the first time. The GLASS priority pathogens *E. coli*, *K. pneumoniae*, *P. aeruginosa*, *A. baumannii* complex, and *Staphylococcus aureus* are considered of great clinical and public health concern worldwide. This study describes the antimicrobial susceptibility profiles of these and other key pathogens isolated from clinical specimens in Timor-Leste between 2020 and 2025, providing a longitudinal overview of national AMR trends following implementation of laboratory automation and quality improvement initiatives during this time.

## Methods

A retrospective descriptive analysis of AST results for WHO priority pathogens isolated from clinical samples submitted to the LoH between June 2020 and January 2025 was performed. Samples were eligible if received from the neighbouring referral hospital, Hospitál Nacionál Guido Valadares (HNGV) in Dili, the five municipality referral hospitals (Oecusse, Maliana, Maubisse, Baucau, and Suai), or Community Health Centres (CHC) across the country, and if laboratory testing confirmed a WHO priority pathogen. All isolates were identified and further tested as part of clinical care, and samples included both sterile and non-sterile sites. The WHO GLASS priority pathogens *E. coli, K. pneumoniae, P. aeruginosa, A. baumannii complex,* and *S. aureus* were the focus of the analysis, chosen as they were among the most common clinical isolates in Timor-Leste and susceptibility testing were routinely performed at the LoH. Other frequently isolated organisms of clinical relevance (*Enterobacter cloacae* complex, *Enterococcus faecalis* and *Haemophilus influenzae*) were included in the aggregated antibiogram but were not incorporated into species-specific trend analyses, as their lower annual isolate numbers precluded reliable year-on-year trend estimation. Trend analyses were therefore restricted to the five WHO GLASS priority pathogens, for which sufficient isolates were available across all years.

**Fig. 1:**
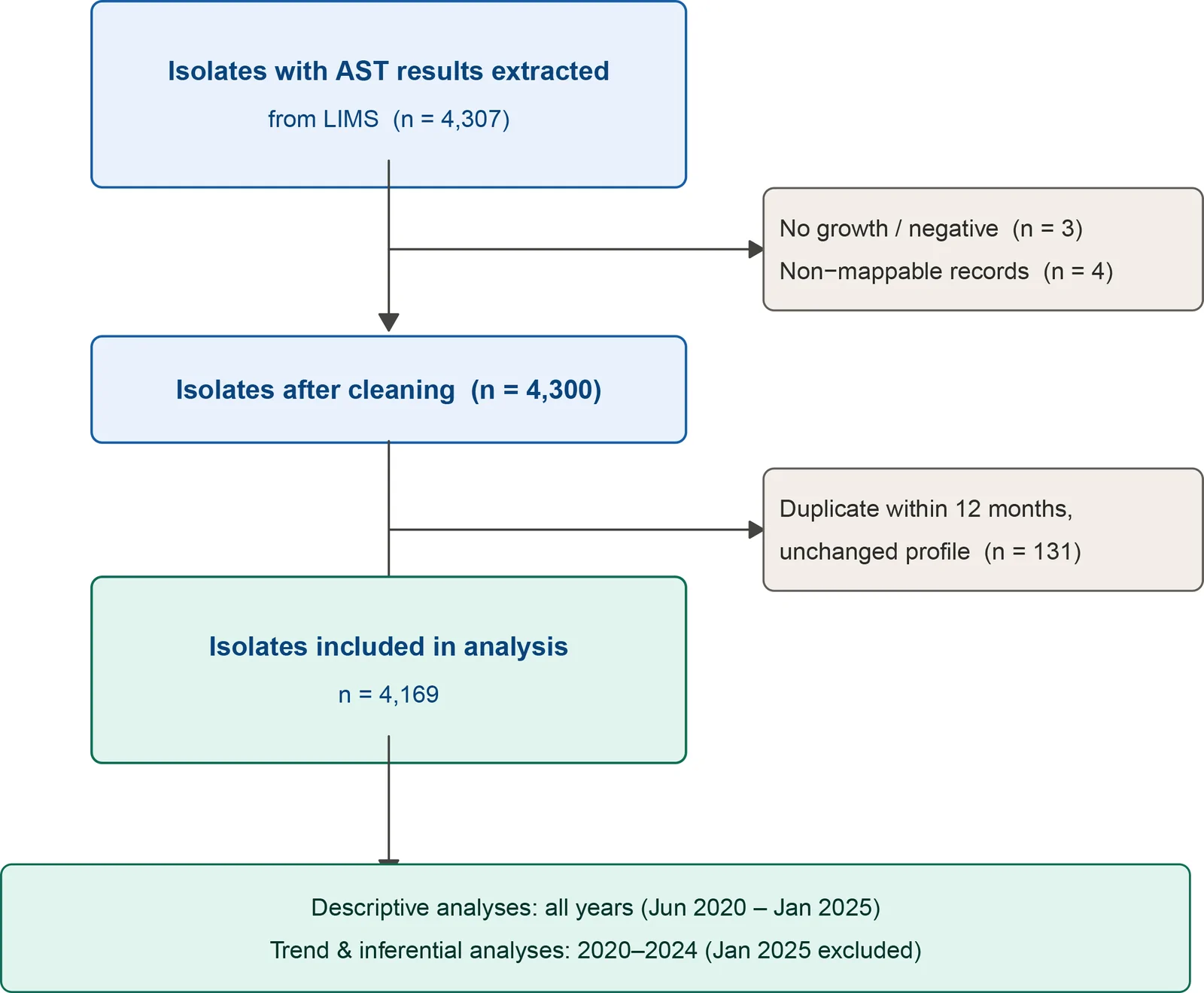
**Flow of bacterial isolates through the study.** Records were extracted from the laboratory information management system as isolates with antimicrobial susceptibility results. Duplicate isolates of the same organism from the same patient within 12 months, where the susceptibility profile was unchanged, were excluded. Descriptive analyses included all 4169 isolates (June 2020–January 2025); trend and inferential analyses were restricted to 2020–2024, with January 2025 excluded.

Data extracted from the LIMS included patient demographics, facility information, and all associated microbiology and antimicrobial susceptibility results. Patient sex was recorded as captured in the LIMS. Data on race or ethnicity were not collected, as the study used routine laboratory records that do not capture this information; the population served is predominantly Timorese. Cultures reported as no growth, negative, or as contaminants were excluded at the point of extraction; a small number of organisms occasionally regarded as contaminants but recovered from sterile sites were retained as potential pathogens, and these constituted 0.8% (32/4169) of isolates, none of which were among the organisms analysed in detail. From 4307 isolates with antimicrobial susceptibility results extracted from the LIMS, 7 were removed during data cleaning (3 reported as no growth or negative, 4 with non-mappable test or result fields) and 131 were excluded as duplicate isolates of the same organism from the same patient within 12 months. Repeat isolates were retained only where the susceptibility profile differed, representing a distinct resistance phenotype, yielding 4169 isolates for analysis (Fig. 1).

All laboratory testing and AST interpretation was carried out as per local standard operating procedures (SOP) based on international guidelines such as those developed by the European Committee on Antimicrobial Susceptibility Testing (EUCAST). Breakpoint tables were updated annually in line with each year's EUCAST revision, and isolates were interpreted using the version current at the time of testing. Following primary culture of clinical specimens on solid agar, bacterial identification was performed by Matrix-Assisted Laser Desorption/Ionization Time-of-Flight (MALDI-ToF, Bruker Daltonik, Bremen, Germany). Antimicrobial susceptibility testing was performed using the BD Phoenix M50 automated system (Becton Dickinson, Berks, UK) for Enterobacterales, *P. aeruginosa*, *A. baumannii* complex, *S. aureus*, and *E. faecalis*. Disc diffusion was used for fastidious organisms not reliably tested on the automated platform, specifically *H. influenzae*. Methicillin resistance was identified phenotypically using cefoxitin as the primary marker, in accordance with EUCAST guidance. Cloxacillin and cefoxitin results were highly concordant where both were recorded (849/852 isolates, 99.6%). As cefoxitin results were incompletely captured in the LIMS in the earlier years of the study, cloxacillin was used as the methicillin-resistance marker for the time-trend analysis, while cefoxitin was used for the cross-sectional analyses by sex, age, specimen type, and care setting. Given their near-perfect concordance, the two markers are considered equivalent for this purpose. Antibiotics were selected for each organism on the basis of clinical relevance and intrinsic resistance. After antibiotic interpretation all relevant results were entered into the laboratory information management system (SchuyLab CGM LIMS).

During the study period, the LoH participated in two external quality assurance (EQA) programmes to ensure the accuracy and reliability of diagnostic testing. The first, coordinated by the Pacific Pathology Training Centre (PPTC) in New Zealand, focused on bacterial culture, identification, and antimicrobial susceptibility testing. LoH also participated in the EQAsia program, supported by the UK Fleming Fund and implemented by the Technical University of Denmark (DTU) and the International Vaccine Institute (IVI), which similarly assesses bacterial identification and susceptibility testing performance. Each EQA round comprised a panel of challenge isolates for which participating laboratories reported organism identification and antimicrobial susceptibility results. Performance scores represent the percentage of challenge isolates for which the laboratory returned correct results (correct species identification and correct susceptibility interpretation against the reference result) in each round. The LoH achieved scores exceeding 90% in all PPTC rounds since 2019 and consistently above 80% in EQAsia rounds since 2021.

### Statistical analysis

The proportion of susceptible isolates was calculated for each organism–antibiotic combination, and 95% confidence intervals were determined using the Wilson method. Cochran-Armitage trend test was used, where applicable, to assess changes in antimicrobial susceptibility over time, with p < 0.05 considered statistically significant. Additionally, associations between non-susceptibility and patient sex, age group, specimen type, and care setting for clinically relevant organism–antibiotic combinations were assessed. Non-susceptibility was defined as a result of intermediate or resistant. Associations were tested using Pearson's χ^2^ test, or Fisher's exact test where any expected cell count was below five, with Bonferroni correction for multiple comparisons. Age was categorised as neonate/infant (<1 year), child (1–14 years), or adult (≥15 years). Care setting at the time of sampling was categorised as outpatient, emergency, general inpatient, or intensive care unit (ICU), and isolates referred from other hospitals or with unknown setting were excluded from the care-setting analysis. January 2025 isolates were excluded from all trend and inferential analyses given the single-month sampling window and the influence of an outbreak cluster. Analysis was performed in R version 4.5.2 (R Foundation for Statistical Computing) using RStudio 2026.01.1.

### Ethics statement

Ethical approval was obtained from the Human Research Ethics Committee of the Northern Territory Department of Health and Menzies School of Health Research (HREC-2024-4834), and the Instituto Nacional de Saúde Pública de Timor-Leste Research Ethics and Technical Committee (1362/INSP-TL/UEPD-AL/IX/2024). A waiver of individual informed consent was granted by both approving committees, as the study used existing de-identified routine diagnostic laboratory data collected as part of clinical care.

### Role of the funding source

The funder had no role in study design, data collection, analysis, interpretation, or writing of the report. The corresponding author had full access to all data and final responsibility for the decision to submit.

## Results

Between June 2020 and January 2025, a total of 4169 bacterial isolates were included. Most of these were from patients older than 15 years (63%, n = 2605) (Table 1). More positive cultures were from males (52%, n = 2170) and over half of all positive cultures were collected from inpatients admitted to a ward or the Intensive Care Unit (ICU) at HNGV (58%, n = 2432).

**Table 1:** Demographic and specimen characteristics of bacterial isolates, June 2020–January 2025.

Variable	Category	2020 (Jun–Dec)	2021	2022	2023	2024	2025 (Jan only)	Total
Sex	Male	115 (44)	349 (55)	485 (51)	487 (50)	655 (54)	79 (61)	2170 (52)
	Female	127 (49)	271 (43)	465 (48)	489 (50)	540 (45)	51 (39)	1943 (47)
	Unknown	17 (7)	11 (2)	11 (1)	3 (0)	11 (1)	0 (0)	56 (1)
Age group	≥15 years	160 (62)	444 (70)	610 (64)	584 (60)	740 (61)	67 (52)	2605 (63)
	<15 years	81 (31)	160 (25)	335 (35)	385 (39)	461 (38)	61 (47)	1483 (36)
	Unknown	18 (7)	27 (4)	16 (2)	10 (1)	8 (1)	2 (2)	81 (2)
Location	Inpatient (HNGV)	135 (52)	358 (57)	525 (55)	462 (47)	611 (51)	48 (37)	2139 (51)
	Outpatient and CHC	50 (19)	159 (25)	236 (25)	241 (25)	250 (21)	28 (22)	964 (23)
	Emergency (HNGV)	13 (5)	46 (7)	96 (10)	142 (15)	143 (12)	28 (22)	468 (11)
	Intensive care unit (HNGV)	6 (2)	40 (6)	38 (4)	43 (4)	144 (12)	22 (17)	293 (7)
	Referral hospitals	11 (4)	18 (3)	59 (6)	83 (9)	52 (4)	3 (2)	226 (5)
	Unknown	44 (17)	10 (2)	7 (1)	8 (1)	9 (1)	1 (1)	79 (2)
Sample type	Blood culture	70 (27)	179 (28)	330 (34)	366 (37)	529 (44)	75 (58)	1549 (37)
	Wound/abscess	90 (35)	176 (28)	258 (27)	267 (27)	327 (27)	23 (18)	1141 (27)
	Urine	55 (21)	122 (19)	143 (15)	122 (13)	130 (11)	3 (2)	575 (14)
	Sputum	15 (6)	74 (12)	115 (12)	90 (9)	121 (10)	18 (14)	433 (10)
	Ear/Nose/Throat	13 (5)	38 (6)	76 (8)	88 (9)	52 (4)	10 (8)	277 (7)
	Urogenital	4 (2)	14 (2)	6 (1)	21 (2)	22 (2)	0 (0)	67 (2)
	Other	6 (2)	17 (3)	17 (2)	5 (1)	13 (1)	1 (1)	59 (1)
	Fluid	1 (0)	5 (1)	11 (1)	10 (1)	6 (1)	0 (0)	34 (1)
	Unknown	3 (1)	4 (1)	2 (0)	8 (1)	6 (1)	0 (0)	23 (1)
	Faeces	2 (1)	2 (0)	2 (0)	2 (0)	3 (0)	0 (0)	11 (0)

Data are n (%). Percentages are column percentages within each year and may not sum to 100 owing to rounding. CHC = community health centre. HNGV=Hospital Nacional Guido Valadares. ICU = intensive care unit. LoH=Laboratory of Health.

**Fig. 2:**
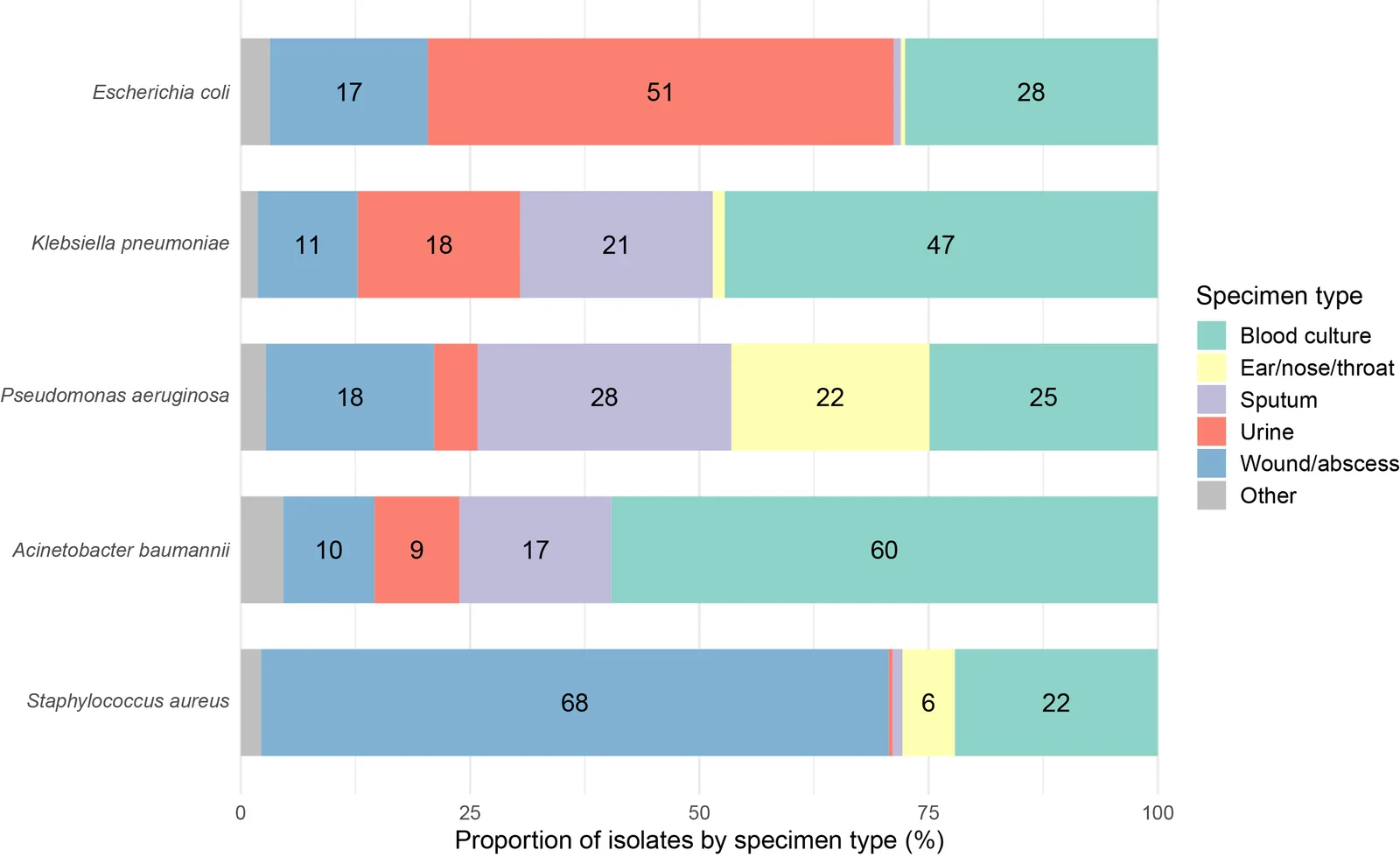
**Distribution of specimen sources by organism.** ‘Other’ includes body fluid, cerebrospinal fluid, intravascular catheter tips, urogenital swabs and stool samples.

Of the 4169 bacterial isolates included in the analysis, 65% (n = 2699) were Gram-negative. The most common organism was *S. aureus* (23%, n = 948), followed by *E. coli* (15%, n = 639), *P. aeruginosa* (13%, n = 546) and *K. pneumoniae* (13%, n = 542). The most common sample type was blood culture (37%, n = 1549), followed by wound/pus (27%, n = 1141) and urine (14%, n = 575). Additionally, specific organisms were more commonly isolated from particular samples: *K. pneumoniae* (256/542, 47%), *S. aureus* (209/948, 22%) and *E. coli* (173/639, 27%) were commonly isolated from blood cultures; *S. aureus* from wound/pus samples (646/948, 68%); and *E. coli* from urine (319/639, 50%) (Fig. 2).

Antimicrobial susceptibility data are summarised in Table 2. Among Enterobacterales, *E. coli* (n = 639) and *K. pneumoniae* (n = 542) showed low susceptibility to amoxicillin–clavulanate (25% and 22%, respectively) and third-generation cephalosporins, with ceftriaxone susceptibility of 65% in *E. coli* and 25% in *K. pneumoniae*. *E. cloacae* (n = 144) demonstrated high susceptibility to amikacin (98%) and meropenem (98%), but reduced susceptibility to ciprofloxacin (48%) and gentamicin (58%). *A. baumannii* (n = 152) displayed extensive resistance, with 40% susceptibility to gentamicin and 60% to meropenem. *P. aeruginosa* (n = 546) demonstrated high susceptibility to amikacin (97%), meropenem (92%) and ciprofloxacin (84%), with reduced susceptibility (78%) to piperacillin–tazobactam. Among Gram-positive organisms, *S. aureus* (n = 948) showed 83% susceptibility to cefoxitin, consistent with methicillin-susceptible *S. aureus* (MSSA) predominance, and complete susceptibility to vancomycin (100%). *E. faecalis* (n = 109) was also uniformly susceptible to vancomycin (100%) but less commonly susceptible to ampicillin (59%), while *H. influenzae* (n = 95) showed unexpectedly low susceptibility to amoxicillin–clavulanate (62%) and 68% to tetracycline.

**Table 2:** Antibiogram of clinically significant organisms isolated at LoH, June 2020–January 2025.

Values are percent susceptible (%S) with the number of isolates tested (n) shown beneath. Cell shading indicates susceptibility: green ≥80%, yellow 60–79%, red <60%. Combinations reflecting intrinsic resistance were not reported. For *S. aureus*, β-lactam susceptibility is inferred from cefoxitin; individual cephalosporins and carbapenems are not reported separately, and cefoxitin (FOX) was used as the marker for methicillin susceptibility. Antibiotics are grouped by class. **Penicillins:** PEN, benzylpenicillin; AMP, ampicillin; AMC, amoxicillin–clavulanate; TZP, piperacillin–tazobactam. **Cephalosporins:** CZO, cefazolin; CEP, cefalothin; CAZ, ceftazidime; CRO, ceftriaxone; FEP, cefepime; FOX, cefoxitin. **Carbapenems:** IPM, imipenem; MEM, meropenem. **Fluoroquinolones:** CIP, ciprofloxacin; LVX, levofloxacin. **Aminoglycosides:** GEN, gentamicin; AMK, amikacin. **Folate pathway inhibitors:** SXT, trimethoprim–sulfamethoxazole. **Other:** NIT, nitrofurantoin; CLI, clindamycin; ERY, erythromycin; DOX, doxycycline; TCY, tetracycline; VAN, vancomycin.

**Fig. 3:**
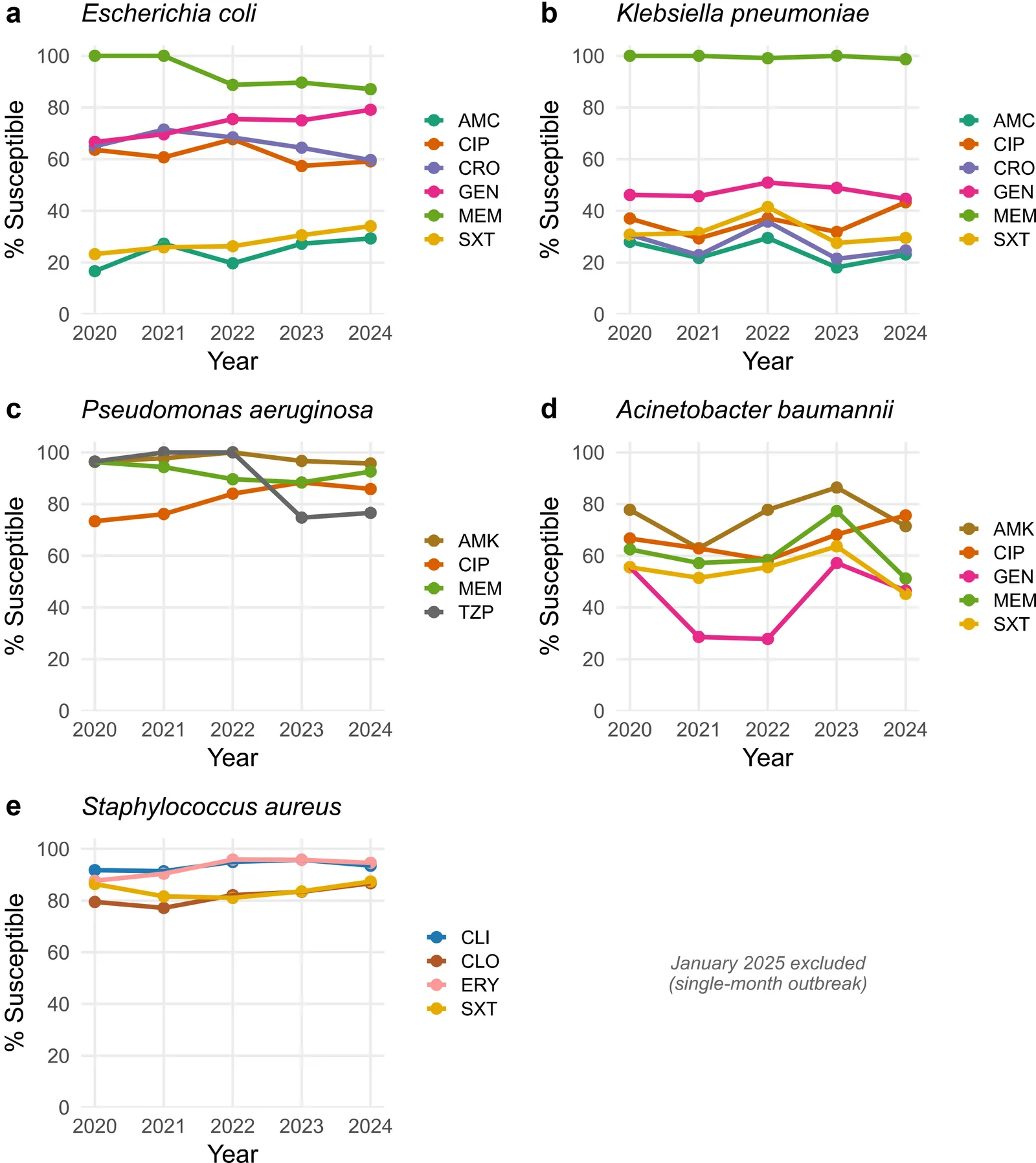
**Trends in antimicrobial susceptibility among GLASS priority organisms, 2020–2024.** Each panel shows the percentage of isolates susceptible to selected antimicrobials by year for (a) *Escherichia coli*, (b) *Klebsiella pneumoniae*, (c) *Pseudomonas aeruginosa*, (d) *Acinetobacter baumannii*, and (e) *Staphylococcus aureus*. January 2025 is excluded. AMK = amikacin. AMC = amoxicillin–clavulanate. CIP = ciprofloxacin. CRO = ceftriaxone. GEN = gentamicin. MEM = meropenem. SXT = trimethoprim–sulfamethoxazole. TZP = piperacillin–tazobactam. CLI = clindamycin. CLO = cloxacillin. ERY = erythromycin.

Time-trend analysis identified a single susceptibility trend that remained statistically significant after correction for multiple comparisons: meropenem in *E. coli*, which declined from 100% susceptible (33/33) in 2020 to 87.1% (155/178) in 2024 (Cochran–Armitage p = 0.0002; Bonferroni-adjusted p = 0.006; Fig. 3), indicating emerging carbapenem resistance. No other trend remained statistically significant after adjustment across the 25 organism–antibiotic combinations tested. Several uncorrected trends were apparent but did not survive correction for multiple testing. These included declining ceftriaxone susceptibility in *E. coli* (65.0%–59.7%; raw p = 0.06, adjusted p = 1.00) and a reduction in piperacillin–tazobactam susceptibility in *P. aeruginosa* (96.4%–76.6%; raw p = 0.012, adjusted p = 0.31). Improving trends not surviving correction were observed for gentamicin susceptibility in *E. coli* and cloxacillin and erythromycin susceptibility in *S. aureus* (all adjusted p > 0.4). *K. pneumoniae* meropenem susceptibility remained ≥98% throughout 2020–2024, with no statistically significant trend over this period (p = 0.28). The apparent fall to 50% in January 2025 was driven by a single-month outbreak cluster of carbapenem-resistant *K. pneumoniae* and is shown descriptively only (Supplementary Data Table 1), and a sensitivity analysis including January 2025 is provided to illustrate its effect. Susceptibility in *A. baumannii* remained low but without significant temporal trend for any agent tested.

Susceptibility varied by sex and age group for several organism–antibiotic combinations which was statistically significant (Supplementary Data Tables 2 and 3). Ceftriaxone susceptibility in *E. coli* was lower in males than females (47% vs. 77%; adjusted p < 0.0001), as was meropenem susceptibility (87% vs. 94%; adjusted p = 0.014). The difference in ceftriaxone susceptibility persisted when restricted to urinary isolates (males 46%, 44/96; females 80%, 175/218), indicating it was not explained by differences in specimen source. In *K. pneumoniae*, ceftriaxone susceptibility differed by age group, being lowest among children (13%, 8/61) and neonates/infants (18%, 18/98) compared with adults (31%, 108/343; adjusted p = 0.019). Methicillin susceptibility in *S. aureus* also differed by age group, being lower among adults (80%, 352/440) than children (89%, 210/235) or neonates/infants (88%, 190/217; adjusted p = 0.028). No statistically significant differences were observed for the remaining organism–antibiotic combinations after correction for multiple testing.

Susceptibility differed by specimen type which was statistically significant for *E. coli* meropenem (adjusted p = 0.038), with bloodstream isolates showing markedly lower carbapenem susceptibility (84%, 138/164) than urinary (94%, 298/317) or wound/pus isolates (95%, 103/108) (Supplementary Data Table 4). A consistent pattern of lower susceptibility among bloodstream isolates was observed for *K. pneumoniae* ceftriaxone (21% vs. 26–40% in other sites) and *S. aureus* methicillin susceptibility (80% vs. 85%), although these did not reach significance after correction for multiple testing. No other statistically significant differences by specimen type were identified. Comparisons involving small denominators (e.g., *E. coli* sputum, *S. aureus* urine; each n < 5) were too small to yield robust estimates or support formal statistical testing.

**Fig. 4:**
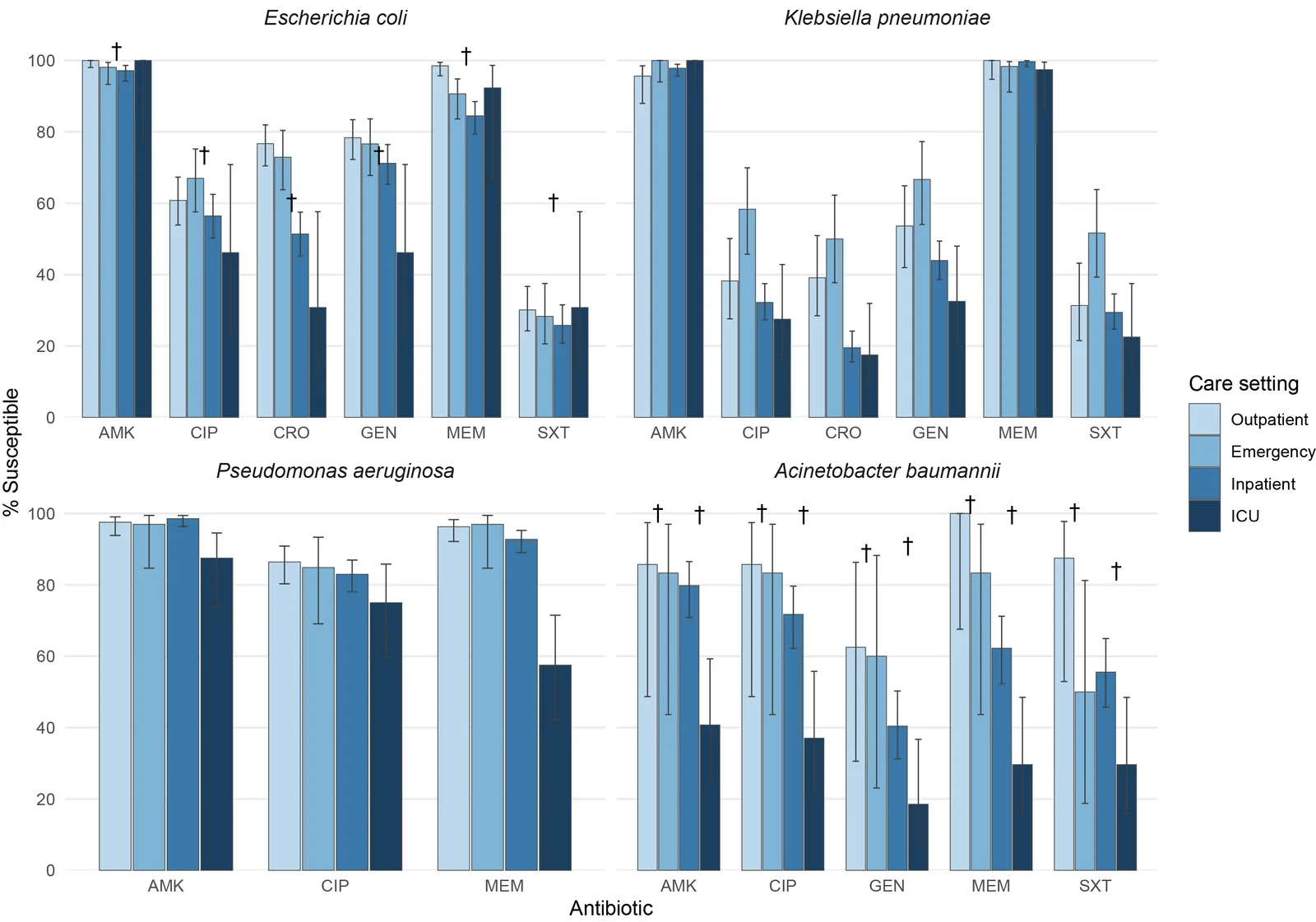
**Antimicrobial susceptibility by care setting for four Gram-negative organisms, 2020–2024.** Bars show the percentage of isolates susceptible to each antibiotic within each care setting; error bars are 95% Wilson confidence intervals. Daggers (†) mark estimates based on fewer than 20 isolates, which should be interpreted with caution. Isolates from other referring hospitals or with unknown setting, and all January 2025 isolates, were excluded. AMK = amikacin. CIP = ciprofloxacin. CRO = ceftriaxone. GEN = gentamicin. MEM = meropenem. SXT = trimethoprim–sulfamethoxazole.

Health care setting was the strongest determinant of susceptibility, with five of seven tested combinations statistically significant after correction (Supplementary Data Table 5, Fig. 4). Ceftriaxone susceptibility decreased across settings from outpatient through emergency and general inpatient care to the ICU for both *E. coli* (77%, 73%, 52% and 31%, 4/13 respectively; adjusted p = 0.0007) and *K. pneumoniae* (39%, 50%, 20% and 18%, 7/40; adjusted p < 0.0001). Meropenem susceptibility was also associated with care setting, for *A. baumannii* it fell from 100% (outpatient) to 30% (8/27) in ICU (adjusted p = 0.004), for *P. aeruginosa* it was high and similar across outpatient, emergency and inpatient settings (93–97%) but markedly reduced in ICU (58%, 23/40; adjusted p = 0.0007), and for *E. coli* it decreased from 99% (outpatient) to 85% among general inpatients (adjusted p = 0.0007), with the ICU estimate based on too few isolates (n = 13) to interpret reliably. No statistically significant association was observed for *K. pneumoniae* meropenem susceptibility, where carbapenem susceptibility was uniformly high, or for *S. aureus* methicillin susceptibility.

On direct comparison of ICU with general inpatient isolates, ceftriaxone susceptibility difference was not statistically significant for either *E. coli* (31% vs. 52%; p = 0.17) or *K. pneumoniae* (18% vs. 20%; p = 1.00), indicating that for these Enterobacterales reduced susceptibility is already established at the general ward level rather than being distinctly ICU-associated. The pronounced ICU reduction was instead confined to carbapenem susceptibility in the non-fermenting organisms *A. baumannii* and *P. aeruginosa*.

Third-generation cephalosporin resistance was common among Enterobacterales isolates. Using ceftriaxone resistance as a phenotypic indicator, 35% of *E. coli* (220/631) and 75% of *K. pneumoniae* (406/541) were non-susceptible, indicative of potential ESBL production. Ceftriaxone non-susceptibility was also high in *E. cloacae* complex (76%, 109/144). However, ESBL status was not confirmed phenotypically (e.g., by combination-disk testing) or genotypically in this study, so these figures should be interpreted as presumptive. Carbapenem resistance, where present, may additionally reflect carbapenemase production, which can independently confer ceftriaxone resistance. The proportion of *S. aureus* isolates that were methicillin-resistant *S. aureus* (MRSA) decreased from 20.5% in 2020 to 13.2% in 2024. MRSA prevalence in the first month of 2025 was 22.2% (n = 6/27), although this single-month estimate is based on few isolates and should interpreted with caution.

**Fig. 5:**
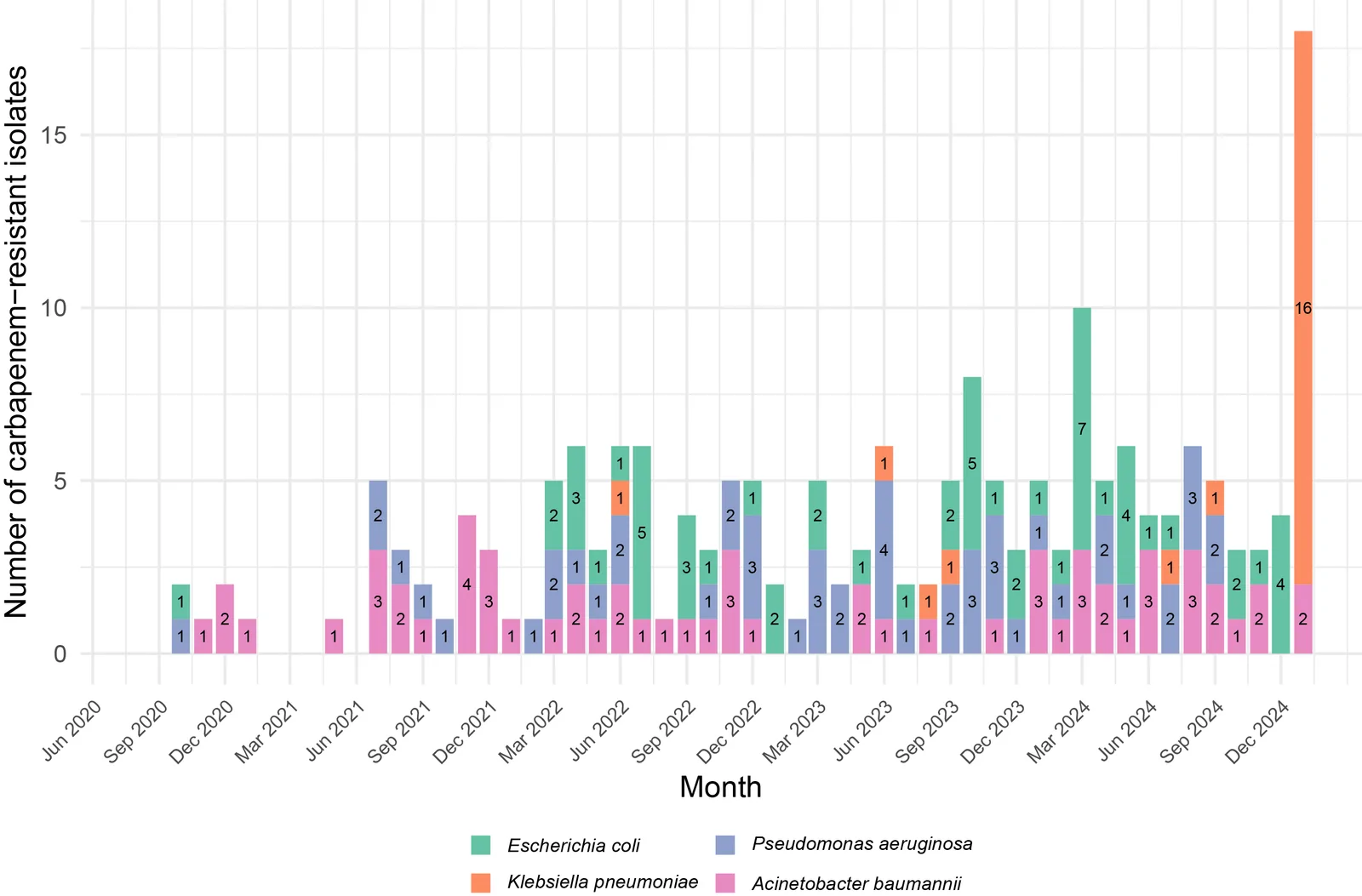
**Monthly counts of carbapenem-resistant isolates by organism, June 2020–January 2025.** Each bar shows the number of carbapenem-resistant isolates identified per month, coloured by organism. The January 2025 peak corresponds to a cluster of carbapenem-resistant *Klebsiella pneumoniae* associated with a paediatric intensive care unit outbreak.

A total of 235 carbapenem-resistant isolates were identified from clinical samples over the study period, of which 29% (n = 61) were *A. baumannii* complex and 23% (n = 54) were *E. coli*. Meropenem resistance in *A. baumannii* complex was detected throughout the study period and ranged from 23% to 49%. In *E. coli*, meropenem resistance increased from 0% (0/33) in early study years to 13% (23/178) in 2024. A cluster of carbapenem-resistant *K. pneumoniae* isolates was identified in January 2025 (n = 16) associated with a Paediatric Intensive Care Unit outbreak (Fig. 5).

## Discussion

Analysis of AMR data from 2020 to 2025 in Timor-Leste demonstrates high levels of resistance among GLASS pathogens, especially among Enterobacterales which exhibited low susceptibility to third-generation cephalosporins and increasing resistance to carbapenems. In *E. coli* and *K. pneumoniae*, this pattern is consistent which could be due to ESBL production, although ESBL status was not confirmed by phenotypic or genotypic methods and ceftriaxone resistance is a non-specific marker. Ceftriaxone resistance in *E. cloacae* complex is more likely attributable to inducible AmpC β-lactamase than to ESBL production and is not interpreted as an ESBL indicator here.

WHO-GLASS data from Indonesia, Malaysia, the Philippines and other regional countries in 2018 reported ceftriaxone resistance ranging from 25 to 82% in *E. coli* and 33–80% in *K. pneumoniae.*^14^ In the neighbouring Pacific region, availability of AMR data is limited, however studies indicate ESBL rates in *K. pneumoniae* of 3.4% in New Caledonia and 22% in Fiji, with an estimated overall 29% of Enterobacterales exhibiting ESBL phenotypes in some settings.^15^, ^16^, ^17^ These similarities indicate a regional pattern of high ESBL prevalence in healthcare settings in LMIC. The high ceftriaxone resistance observed (75% in *K. pneumoniae*) substantially exceeds rates reported regionally and should be interpreted in the context of the sampling frame. As most isolates were derived from a tertiary referral centre, our care-setting analysis also demonstrated higher resistance among inpatient and ICU isolates than outpatient isolates, these figures likely reflect a hospital-weighted, high-acuity population rather than the national community burden. Caution is therefore warranted in extrapolating these rates to the general Timor-Leste population. Clusters of third-generation cephalosporin- and carbapenem-resistant *K. pneumoniae* were noted in paediatric and intensive care units during the study period, most notably the carbapenem-resistant cluster of January 2025. Because such nosocomial clusters concentrate resistant isolates within short periods, they can influence aggregate resistance proportions; this is reflected in our sensitivity analysis showing that the apparent 2025 decline in *K. pneumoniae* meropenem susceptibility was driven entirely by that single outbreak.

Previous publications outlining the burden of AMR in Timor-Leste are limited, and no reports identified carbapenem resistance in Enterobacterales and their emergence represents an urgent clinical challenge.^9^^,^^10^^,^^18^^,^^19^ Previously reported carbapenem resistance was confined to *A. baumannii*, with the first NDM-1 producing isolate reported in 2020.^20^ Repeated isolation of meropenem-resistant *A. baumannii* throughout the study period suggests that this organism may now be endemic within HNGV. As carbapenemase production and other resistance mechanisms were not characterised phenotypically or genotypically in this study, we cannot confirm the underlying mechanism or demonstrate clonal or plasmid-mediated spread. The concurrent emergence of carbapenem resistance in other Gram-negative species is nonetheless consistent with increasing hospital-based selection pressure. Susceptibility of *A. baumannii* to other antimicrobials was low, indicating established multidrug resistance in this organism in hospital settings. Rising meropenem resistance in *E. coli* is of concern, with no resistant isolates documented in earlier years increasing to a prevalence of 13% in 2024. This trend is observed across Southeast Asia with regional reports of increasing carbapenem-resistance among Enterobacterales.^21^ These findings emphasise the need for infection prevention and control measures targeting high-risk inpatient areas to preserve antibiotic efficacy. Given the limited therapeutic options available for treatment of carbapenem resistant organisms in Timor-Leste, access to newer agents such as ceftazidime–avibactam or meropenem–vaborbactam may become increasingly important. However, the introduction of such reserve agents must be accompanied by parallel strengthening of infection prevention and control and antimicrobial stewardship, including regulatory frameworks governing their use, to avoid further amplification of resistance. The overall cefoxitin susceptibility of around 83% in *S. aureus* (MRSA -17%) and the similar rates observed in inpatient and outpatient settings suggest that, while MRSA remains an important pathogen, it is not disproportionately concentrated in hospital-based care in this setting. The proportion of isolates originating from the ICU increased substantially over the study period (from 2% in 2020 to 12% in 2024). As ICU isolates carry higher resistance, this shift in case-mix may account for part of the apparent temporal increase in resistance and observed trends should therefore be interpreted with this changing denominator in mind.

The low susceptibility to amoxicillin–clavulanate among *H. influenzae* isolates in this study (62%) is unexpected, as β-lactamase–producing strains are usually susceptible. Without β-lactamase data, this pattern is most consistent with β-lactamase–negative ampicillin-resistant (BLNAR) or β-lactamase-positive, amoxicillin–clavulanate-resistant (BLPACR) phenotypes, which reduce the activity of both ampicillin and amoxicillin–clavulanate and are increasingly reported across the Asia–Pacific.^22^ *H. influenzae* was identified by MALDI-ToF and susceptibility tested against the routine SOP panel (penicillin screen, ampicillin, amoxicillin–clavulanate, ceftriaxone, co-trimoxazole, and tetracycline). Ceftriaxone susceptibility was uniformly high (100%, 95/95) and the remaining results were internally consistent. We cannot exclude that laboratory factors such as testing medium or incubation conditions could have contributed to incorrect reporting of amoxicillin–clavulanate susceptibility specifically, however as the remaining results were unchanged we retained the organism in the aggregate antibiogram while interpreting its amoxicillin–clavulanate susceptibility with caution. Misidentification is unlikely given the use of MALDI-ToF and the uniformly high ceftriaxone susceptibility consistent with correctly identified *H. influenzae*. These findings emphasise the importance of β-lactamase testing and confirmatory identification, and of ongoing monitoring of *H. influenzae* susceptibility in Timor-Leste.

Inferential analysis revealed associations between resistance patterns and patient demographics that extend beyond aggregate resistance proportions. Male sex was associated with higher ceftriaxone and meropenem non-susceptibility in *E. coli*, with the ceftriaxone difference persisting within urinary isolates and therefore not attributable to differences in specimen source. The magnitude of this difference (54% vs. 20% in urinary isolates) is notable and suggests a true association rather than a sampling artifact. While the absence of clinical metadata precludes definitive explanation, this pattern may reflect greater cumulative healthcare exposure, higher rates of comorbidity or recurrent urinary tract infection, differences in prior antibiotic use or underlying urological pathology among male patients. This finding warrants further investigation incorporating clinical and prescribing data to clarify the underlying drivers in the Timor-Leste context.

Age group also showed statistically significant associations with resistance, though in opposing directions for different organisms. Ceftriaxone non-susceptibility in *K. pneumoniae* was highest among children and neonates/infants (87% and 82%, respectively) compared with adults (69%), consistent with the concentration of paediatric *K. pneumoniae* isolates in neonatal and paediatric intensive care settings, where nosocomial transmission and antibiotic selection pressure are greatest. This age pattern most likely reflects the care setting in which these isolates arise rather than an intrinsic biological effect of age, and aligns with the *K. pneumoniae* clusters identified in these units during the study period. By contrast, methicillin resistance in *S. aureus* was higher among adults (20%) than children or neonates/infants (11% and 12%), a pattern more consistent with the adult risk factors classically associated with MRSA, including prior hospitalisation, chronic wounds, indwelling devices, and repeated antibiotic exposure. Together, these contrasting age associations underscore that demographic patterns in resistance are driven largely by the clinical contexts in which different organisms are encountered, reinforcing the need for setting-specific infection prevention and stewardship strategies.

Carbapenem non-susceptibility in *E. coli* was higher in bloodstream isolates than in isolates from other sites (adjusted p = 0.038), with a consistent but statistically non-significant trend toward higher resistance in bloodstream isolates across other organism–antibiotic combinations. This likely reflects the predominance of inpatient, hospital-associated infection among bloodstream isolates, where carbapenem exposure and transmission of resistant strains are concentrated, and is consistent with the higher resistance observed among inpatient isolates elsewhere in this study.

The persistently high burden of AMR observed in this study likely reflects broader systemic challenges in implementing antimicrobial stewardship and infection control measures in Timor-Leste. The absence of established frameworks to regulate access to reserve antibiotics has contributed to widespread use of agents such as carbapenems and third-generation cephalosporins, often without formal prescribing oversight. While an Antimicrobial Stewardship Committee is now being established at HNGV, coordinated monitoring of antimicrobial use was still in development during the study period.^23^ High turnover among hospital staff, combined with limited resources for ongoing training, further complicates consistent adherence to infection prevention and control protocols. These issues are common across health systems in resource-limited settings and highlight the importance of sustained investment in antimicrobial stewardship, workforce capacity, and laboratory strengthening initiatives.

There are several limitations to this study. Clinical sample collection was determined by treating clinicians, and the patterns observed may have been influenced by case mix and referral practices, including disruptions associated with the coronavirus disease 2019 (COVID-19) pandemic. As the data were obtained through routine diagnostic testing at the national referral laboratory, they reflect the microbiological and antimicrobial susceptibility testing capacity available at the time, which varied across the study period. Importantly, the study period coincided with substantial expansion of diagnostic capacity, including the introduction of MALDI-ToF identification, automated susceptibility testing, a LIMS, and a marked increase in specimen submissions. Some of the apparent increase in resistance detection may therefore reflect improved identification and greater surveillance sensitivity rather than true epidemiological emergence alone. Because isolates were interpreted using the EUCAST breakpoints current in each year, revisions to breakpoints over the study period may have contributed to apparent temporal changes in susceptibility independent of true changes in resistance, particularly for organism–antibiotic combinations where breakpoints were redefined. Occasional interpretative error may also have affected classification. Clinical information was limited, and there were no data on treatment, comorbidities, or patient outcomes, restricting the ability to contextualise resistance patterns or assess clinical impact. It was also not possible to reliably distinguish community-acquired from hospital-acquired infections, although inpatient samples are likely to include a substantial proportion of hospital-associated infections. As most isolates originated from HNGV, findings may not represent other healthcare facilities in Timor-Leste, and expanded surveillance across regional hospitals is required to understand the broader national AMR epidemiology.

Timor-Leste has made considerable progress in strengthening diagnostic laboratory capacity to generate reliable AMR surveillance data. Despite persistent challenges, the establishment of systematic diagnostic system and data reporting processes has enabled the identification of key resistance trends, including the emergence of carbapenem-resistant Enterobacterales and the probability of ESBL-producing organisms. These findings highlight the presence of clinically important resistance phenotypes aligned with WHO priority pathogens and emphasise the need for ongoing investment in diagnostic capacity, infection prevention, and antimicrobial stewardship. Strengthened laboratory systems, including LIMS, provide a critical evidence base to guide empirical treatment protocols, inform national antimicrobial policy, and support Timor-Leste’s continued integration into regional and global AMR surveillance networks.

## Contributors

Conceptualisation: TO, CDCM, HS, IM, JY, JRF; Data curation: TO, CDCM, CO, MS, JB, VDC, RA; Formal analysis: TO, ADKD, HS, JRF, JY, RA; Funding acquisition: KC, JY, JRF; Investigation: TO, CDCM, CO, MS, JB, VDC, RA, EA, LT; Methodology: TO, CDCM; Project administration: KC; Supervision: HS, JRF, ESDS, DDCC, AJPT, LA, NS; Validation: HS, JRF, ADKD, RA, BD, IM; Visualisation: TO; Writing—original draft: TO, CDCM; Writing—review & editing: All authors.

TO and CDCM accessed and verified the underlying data. All authors had access to the data reported in the study and approved the decision to submit for publication.

## Data sharing statement

Data used in the analysis and interpretation of this work are available in the Supplementary Data Tables.

## Declaration of interests

Authors do not have any conflict of interest to declare.

## Appendix A Supplementary data

Supplementary Data Tables 1–5

Translated Abstract
