# Supplementary material for: Antimicrobial susceptibility of priority bacterial pathogens from laboratory samples in Timor-Leste: a retrospective analysis of database (2020–2025)
Source: Lancet Reg Health Southeast Asia. 2026 Aug 20;53:100847. doi: 10.1016/j.lansea.2026.100847 (PMC13521173; doi:10.1016/j.lansea.2026.100847)
Supplement: Supplementary file 1 [file mmc1.docx]

**Supplementary Data**

Contents

[Supplementary Table 1: Cochran–Armitage trend analysis of susceptibility, 2020–2024	2](#_Toc235459907)

[Supplementary Table 2. Susceptibility by sex and age group (2020-2024)	4](#_Toc235459908)

[Supplementary Table 3. Susceptibility by sex and age group (2020-2024)	7](#_Toc235459909)

[Supplementary Table 4. Susceptibility by specimen type for clinically relevant organism–antibiotic combinations, 2020–2024	8](#_Toc235459910)

[Supplementary Table 5. Susceptibility by care setting for clinically relevant organism–antibiotic combinations, 2020–2024	9](#_Toc235459911)

### Supplementary Table 1: Cochran–Armitage trend analysis of susceptibility, 2020–2024

| **Organism** | **Antibiotic** | **2020 %S (n/N)** | **2021 %S (n/N)** | **2022 %S (n/N)** | **2023 %S (n/N)** | **2024 %S (n/N)** | **2025 %S (n/N)** | **C-A p value** | **C-A p (Bonferroni)** |
| --- | --- | --- | --- | --- | --- | --- | --- | --- | --- |
| *Escherichia coli* | AMC | 16·7 (6/36) | 27·3 (30/110) | 19·7 (29/147) | 27·3 (33/121) | 29·3 (39/133) | 22·2 (2/9) | 0·16 | 1·00 |
|  | CIP | 63·6 (28/44) | 60·7 (68/112) | 67·8 (103/152) | 57·4 (78/136) | 59·2 (100/169) | 70·0 (7/10) | 0·34 | 1·00 |
|  | CRO | 65·0 (26/40) | 71·4 (80/112) | 68·4 (104/152) | 64·4 (87/135) | 59·7 (108/181) | 63·6 (7/11) | 0·06 | 1·00 |
|  | GEN | 66·7 (28/42) | 69·6 (78/112) | 75·5 (114/151) | 75·0 (102/136) | 79·1 (144/182) | 90·9 (10/11) | 0·037 | 0·92 |
|  | MEM | 100·0 (33/33) | 100·0 (110/110) | 88·7 (134/151) | 89·6 (121/135) | 87·1 (155/178) | 100·0 (11/11) | 0·0002 | **0·006** |
|  | SXT | 23·3 (10/43) | 25·9 (29/112) | 26·3 (40/152) | 30·6 (41/134) | 34·1 (62/182) | 54·5 (6/11) | 0·049 | 1·00 |
| *Klebsiella pneumoniae* | AMC | 28·0 (7/25) | 21·7 (20/92) | 29·5 (31/105) | 18·1 (21/116) | 23·1 (31/134) | 3·3 (1/30) | 0·51 | 1·00 |
|  | CIP | 37·0 (10/27) | 29·3 (27/92) | 37·1 (39/105) | 31·9 (43/135) | 43·3 (65/150) | 15·6 (5/32) | 0·11 | 1·00 |
|  | CRO | 30·8 (8/26) | 22·8 (21/92) | 35·8 (38/106) | 21·5 (29/135) | 24·7 (37/150) | 3·1 (1/32) | 0·41 | 1·00 |
|  | GEN | 46·2 (12/26) | 45·7 (42/92) | 50·9 (54/106) | 48·9 (66/135) | 44·7 (67/150) | 25·0 (8/32) | 0·78 | 1·00 |
|  | MEM | 100·0 (25/25) | 100·0 (92/92) | 99·1 (105/106) | 100·0 (135/135) | 98·7 (147/149) | 50·0 (16/32) | 0·28 | 1·00 |
|  | SXT | 30·8 (8/26) | 31·5 (29/92) | 41·5 (44/106) | 27·6 (37/134) | 29·5 (44/149) | 12·5 (4/32) | 0·35 | 1·00 |
| *Pseudomonas aeruginosa* | AMK | 96·4 (27/28) | 97·7 (86/88) | 100·0 (125/125) | 96·7 (116/120) | 95·7 (156/163) | 100·0 (18/18) | 0·19 | 1·00 |
|  | CIP | 73·3 (22/30) | 76·1 (67/88) | 84·0 (105/125) | 88·3 (106/120) | 85·8 (139/162) | 100·0 (18/18) | 0·015 | 0·39 |
|  | MEM | 96·3 (26/27) | 94·3 (83/88) | 89·6 (112/125) | 88·3 (106/120) | 92·6 (150/162) | 100·0 (18/18) | 0·53 | 1·00 |
|  | TZP | 96·4 (27/28) | 100·0 (3/3) | 100·0 (7/7) | 74·8 (77/103) | 76·6 (121/158) | 76·5 (13/17) | 0·012 | 0·31 |
| *Acinetobacter baumannii* | AMK | 77·8 (7/9) | 62·9 (22/35) | 77·8 (28/36) | 86·4 (19/22) | 71·4 (30/42) | 57·1 (4/7) | 0·56 | 1·00 |
|  | CIP | 66·7 (6/9) | 62·9 (22/35) | 58·3 (21/36) | 68·2 (15/22) | 75·6 (31/41) | 71·4 (5/7) | 0·21 | 1·00 |
|  | GEN | 55·6 (5/9) | 28·6 (10/35) | 27·8 (10/36) | 57·1 (12/21) | 46·5 (20/43) | 57·1 (4/7) | 0·16 | 1·00 |
|  | MEM | 62·5 (5/8) | 57·1 (20/35) | 58·3 (21/36) | 77·3 (17/22) | 51·2 (22/43) | 71·4 (5/7) | 0·73 | 1·00 |
|  | SXT | 55·6 (5/9) | 51·4 (18/35) | 55·6 (20/36) | 63·6 (14/22) | 45·2 (19/42) | 42·9 (3/7) | 0·61 | 1·00 |
| *Staphylococcus aureus* | CLI | 91·8 (67/73) | 91·3 (116/127) | 95·0 (207/218) | 95·8 (227/237) | 93·5 (244/261) | 100·0 (27/27) | 0·36 | 1·00 |
|  | CLO | 79·5 (58/73) | 77·2 (98/127) | 82·1 (179/218) | 83·4 (196/235) | 86·7 (222/256) | 77·8 (21/27) | 0·019 | 0·48 |
|  | ERY | 87·7 (64/73) | 90·4 (113/125) | 95·9 (209/218) | 95·8 (227/237) | 94·6 (247/261) | 92·6 (25/27) | 0·020 | 0·49 |
|  | SXT | 86·4 (51/59) | 81·6 (102/125) | 81·0 (175/216) | 83·5 (198/237) | 87·4 (228/261) | 88·9 (24/27) | 0·21 | 1·00 |

**Supplementary Table 1.** Values are percent susceptible with counts (n/N) by year. P values are from the Cochran–Armitage test for linear trend, calculated over 2020–2024; January 2025 isolates are shown descriptively but were excluded from trend testing given the single-month sampling window and the influence of an outbreak cluster. Bonferroni-adjusted p values account for the 25 trends tested; bold values are significant (adjusted p<0·05). Cloxacillin was used as the methicillin-resistance marker for the time-trend analysis; cefoxitin (used for the cross-sectional analyses) was incompletely recorded in the earlier study years, and the two markers were 99·6% concordant where both were tested. Trends were tested only where at least three years had testable isolates.

## Supplementary Table 2. Susceptibility by sex and age group (2020-2024)

| **Organism** | **Sex** | **Age group** | **N** | **Ceftriaxone susceptible** | **Meropenem susceptible** | **Cefoxitin susceptible** |
| --- | --- | --- | --- | --- | --- | --- |
| Acinetobacter baumannii | Female | Adult (≥15yr) | 32 | 15/32 (46·9%) | 18/32 (56·2%) | - |
|  | Female | Child (1-14yr) | 16 | 7/16 (43·8%) | 10/16 (62·5%) | - |
|  | Female | Neonate/Infant (<1yr) | 22 | 17/22 (77·3%) | 13/22 (59·1%) | - |
|  | Male | Adult (≥15yr) | 51 | 15/51 (29·4%) | 32/51 (62·7%) | - |
|  | Male | Child (1-14yr) | 12 | 5/12 (41·7%) | 9/12 (75·0%) | - |
|  | Male | Neonate/Infant (<1yr) | 8 | 4/8 (50·0%) | 3/8 (37·5%) | - |
| Escherichia coli | Female | Adult (≥15yr) | 335 | 259/335 (77·3%) | 316/335 (94·3%) | - |
|  | Female | Child (1-14yr) | 23 | 15/23 (65·2%) | 21/23 (91·3%) | - |
|  | Female | Neonate/Infant (<1yr) | 11 | 10/11 (90·9%) | 11/11 (100·0%) | - |
|  | Male | Adult (≥15yr) | 199 | 87/199 (43·7%) | 172/199 (86·4%) | - |
|  | Male | Child (1-14yr) | 22 | 13/22 (59·1%) | 20/22 (90·9%) | - |
|  | Male | Neonate/Infant (<1yr) | 19 | 13/19 (68·4%) | 16/19 (84·2%) | - |
| Klebsiella pneumoniae | Female | Adult (≥15yr) | 151 | 55/151 (36·4%) | 150/151 (99·3%) | - |
|  | Female | Child (1-14yr) | 24 | 1/24 (4·2%) | 24/24 (100·0%) | - |
|  | Female | Neonate/Infant (<1yr) | 45 | 9/45 (20·0%) | 45/45 (100·0%) | - |
|  | Male | Adult (≥15yr) | 186 | 52/186 (28·0%) | 185/186 (99·5%) | - |
|  | Male | Child (1-14yr) | 37 | 7/37 (18·9%) | 37/37 (100·0%) | - |
|  | Male | Neonate/Infant (<1yr) | 52 | 9/52 (17·3%) | 51/52 (98·1%) | - |
| Pseudomonas aeruginosa | Female | Adult (≥15yr) | 137 | 3/137 (2·2%) | 127/137 (92·7%) | - |
|  | Female | Child (1-14yr) | 39 | 0/39 (0·0%) | 36/39 (92·3%) | - |
|  | Female | Neonate/Infant (<1yr) | 27 | 0/27 (0·0%) | 25/27 (92·6%) | - |
|  | Male | Adult (≥15yr) | 237 | 1/237 (0·4%) | 210/237 (88·6%) | - |
|  | Male | Child (1-14yr) | 63 | 1/63 (1·6%) | 61/63 (96·8%) | - |
|  | Male | Neonate/Infant (<1yr) | 19 | 0/19 (0·0%) | 19/19 (100·0%) | - |
| Staphylococcus aureus | Female | Adult (≥15yr) | 210 | - | - | 158/210 (75·2%) |
|  | Female | Child (1-14yr) | 97 | - | - | 86/97 (88·7%) |
|  | Female | Neonate/Infant (<1yr) | 104 | - | - | 93/104 (89·4%) |
|  | Male | Adult (≥15yr) | 225 | - | - | 190/225 (84·4%) |
|  | Male | Child (1-14yr) | 135 | - | - | 121/135 (89·6%) |
|  | Male | Neonate/Infant (<1yr) | 108 | - | - | 92/108 (85·2%) |

**Supplementary Table 2.** Values are n/N (%). Cefoxitin shown for S. aureus only. Proportions are shown for isolates with both sex and age recorded; p values were calculated on all isolates with the relevant variable recorded, so test denominators (N) may differ slightly from the sum of proportions.

## Supplementary Table 3. Susceptibility by sex and age group (2020-2024)

Table 2. Chi-squared/Fisher test results: susceptibility by sex and age group (2020–2024)

| **Organism** | **Comparison** | **Antibiotic** | **N** | **p value** |
| --- | --- | --- | --- | --- |
| Acinetobacter baumannii | Age group | Meropenem | 142 | 1·00 |
|  | Sex | Meropenem | 142 | 1·00 |
| Escherichia coli | Age group | Ceftriaxone | 613 | 1·00 |
|  |  | Meropenem | 613 | 1·00 |
|  | Sex | Ceftriaxone | 616 | **<0·0001** |
|  |  | Meropenem | 616 | **0·014** |
| Klebsiella pneumoniae | Age group | Ceftriaxone | 502 | **0·019** |
|  |  | Meropenem | 502 | 1·00 |
|  | Sex | Ceftriaxone | 501 | 1·00 |
|  |  | Meropenem | 501 | 1·00 |
| Pseudomonas aeruginosa | Age group | Meropenem | 523 | 1·00 |
|  | Sex | Meropenem | 526 | 1·00 |
| Staphylococcus aureus | Age group | Cefoxitin (MRSA) | 892 | **0·028** |
|  | Sex | Cefoxitin (MRSA) | 907 | 0·63 |

**Supplementary Table 3.** Bold p values are statistically significant (p<0·05). Tests were Pearson's chi-squared without continuity correction, or Fisher's exact where any expected cell count was <5. Analyses restricted to 2020-2024. Proportions are shown for isolates with both sex and age recorded; p values were calculated on all isolates with the relevant variable recorded, so test denominators (N) may differ slightly from the sum of proportions. p values are Bonferroni-corrected across all [n] tests.

## Supplementary Table 4. Susceptibility by specimen type for clinically relevant organism–antibiotic combinations, 2020–2024

| **Organism** | **Antibiotic** | **Blood culture** | **Urine** | **Wound/Pus** | **Sputum** | **ENT** | **Test used** | **Raw p** | **Adjusted p** |
| --- | --- | --- | --- | --- | --- | --- | --- | --- | --- |
| *Acinetobacter baumannii* | Meropenem | 48/84 (57%) | 8/14 (57%) | 10/15 (67%) | 18/24 (75%) | - | Chi-squared | 0·42 | 1·00 |
| *Escherichia coli* | Ceftriaxone | 100/164 (61%) | 221/317 (70%) | 68/108 (63%) | 1/4 (25%) | 2/3 (67%) | Fisher | 0·10 | 0·72 |
|  | Meropenem | 138/164 (84%) | 298/317 (94%) | 103/108 (95%) | 4/4 (100%) | 3/3 (100%) | Fisher | 0·005 | **0.038** |
| *Klebsiella pneumoniae* | Ceftriaxone | 49/234 (21%) | 29/95 (30%) | 23/58 (40%) | 28/107 (26%) | 4/7 (57%) | Fisher | 0·011 | 0·08 |
|  | Meropenem | 233/234 (100%) | 95/95 (100%) | 57/58 (98%) | 106/107 (99%) | 7/7 (100%) | Fisher | 0·45 | 1·00 |
| *Pseudomonas aeruginosa* | Meropenem | 120/131 (92%) | 24/26 (92%) | 86/97 (89%) | 133/147 (90%) | 110/112 (98%) | Fisher | 0·041 | 0·29 |
| *Staphylococcus aureus* | Cefoxitin (MRSA) | 159/199 (80%) | 1/4 (25%) | 539/631 (85%) | 8/10 (80%) | 46/51 (90%) | Fisher | 0·012 | 0·08 |

**Supplementary Table 4.** Values are number susceptible / number tested (% susceptible). Intermediate or resistant isolates were counted as non-susceptible. P values from Pearson's chi-squared test, or Fisher's exact test where any expected cell count was <5. Adjusted p values are Bonferroni-corrected across all 7 tests; bold values are significant (adjusted p<0·05). Cells with fewer than five isolates (e.g. E. coli sputum, S. aureus urine) should not be interpreted. The heterogeneous 'Other' category was excluded from analysis. ENT denotes ear/nose/throat specimens. Cefoxitin was used as the marker for methicillin susceptibility in S. aureus. Ceftriaxone was not assessed for A. baumannii or P. aeruginosa. January 2025 isolates were excluded.

## Supplementary Table 5. Susceptibility by care setting for clinically relevant organism–antibiotic combinations, 2020–2024

| **Organism** | **Antibiotic** | **Outpatient** | **Emergency** | **Inpatient** | **ICU** | **Test used** | **Raw p** | **Adjusted p** |
| --- | --- | --- | --- | --- | --- | --- | --- | --- |
| *Acinetobacter baumannii* | Meropenem | 8/8 (100%) | 5/6 (83%) | 62/99 (63%) | 8/27 (30%) | Fisher | 0·0006 | **0·004** |
| *Escherichia coli* | Ceftriaxone | 161/209 (77%) | 78/107 (73%) | 130/252 (52%) | 4/13 (31%) | Fisher | <0·0001 | **0·0007** |
|  | Meropenem | 206/209 (99%) | 97/107 (91%) | 214/252 (85%) | 12/13 (92%) | Fisher | <0·0001 | **0·0007** |
| *Klebsiella pneumoniae* | Ceftriaxone | 27/69 (39%) | 30/60 (50%) | 63/323 (20%) | 7/40 (18%) | Chi-squared | <0·0001 | **<0·0001** |
|  | Meropenem | 69/69 (100%) | 59/60 (98%) | 322/323 (100%) | 39/40 (98%) | Fisher | 0·16 | 1·00 |
| *Pseudomonas aeruginosa* | Meropenem | 158/164 (96%) | 32/33 (97%) | 257/277 (93%) | 23/40 (58%) | Fisher | <0·0001 | **0·0007** |
| *Staphylococcus aureus* | Cefoxitin (MRSA) | 142/162 (88%) | 72/88 (82%) | 411/497 (83%) | 19/24 (79%) | Fisher | 0·40 | 1·00 |

**Supplementary Table 5.** Values are number susceptible / number tested (% susceptible). Isolates of intermediate or resistant category were counted as non-susceptible (i.e. only susceptible isolates contribute to the numerator). Care setting at time of sampling: Outpatient, Emergency, Inpatient (general ward), and ICU. Isolates from other referring hospitals and those with unknown setting were excluded. P values from Pearson's chi-squared test, or Fisher's exact test where any expected cell count was <5. Adjusted p values are Bonferroni-corrected across all 7 tests; bold values are significant (adjusted p<0·05). Cells with small denominators (e.g. E. coli ICU, n=13) should be interpreted with caution. Cefoxitin was used as the marker for methicillin susceptibility in S. aureus. Ceftriaxone was not assessed for A. baumannii or P. aeruginosa. January 2025 isolates were excluded.
