# Supplementary material for: Antimicrobial susceptibility of priority bacterial pathogens from laboratory samples in Timor-Leste: a retrospective analysis of database (2020–2025)
Source: Lancet Reg Health Southeast Asia. 2026 Aug 20;53:100847. doi: 10.1016/j.lansea.2026.100847 (PMC13521173; doi:10.1016/j.lansea.2026.100847)
Supplement: Supplementary file 2 [file mmc2.docx]

***Disclaimer***

***This translation in Tetum was submitted by the authors and we reproduce it as supplied. It has not been peer reviewed. Our editorial processes have only been applied to the original abstract in English, which should serve as reference for this manuscript.***

## Rezumu Badak

**Kontestu**

Rezisténsia antimikrobiál (AMR) hanesan ameasa globál bo’ot ida, no hatodan liu nasaun sira ho rendimentu ki’ik (sigla inglés: LMIC) iha ne'ebé kapasidade vijilánsia nian dalabarak limitadu. Iha Timor-Leste, mikrobiolojia diagnóstiku no kapasidade vijilánsia ameliora ona ho nível konsideravel iha tinan 10 ikus ne’e. Ami nia objetivu atu deskreve padraun suseptibilidade antimikrobiál no tendénsia temporál sira entre patojéniu baktéria prioridade sira iha Timor-Leste entre tinan 2020 no tinan 2025.

**Métodu sira**

Ami analiza retrospektivamente baktéria individual sira ne'ebé signifikativu ho método klínika ne'ebé prosesa iha Laboratóriu Saúde INSP-TL, entre fulan Juñu tinan 2020 no fulan Janeiru tinan 2025. Patójenu prioridade WHO GLASS nian identifika uza MALDI-ToF ho AST automatizadu no interpreta uza padraun EUCAST. Duplikasaun sira iha fulan 12 nia laran ami la inklui. Proporsaun suseptibilidade, tendénsia temporál, no asosiasaun entre AMR no seksu, idade, tipu espesímen, ami análiza ona ho kuidadu.

**Deskobrimentu sira**

Husi bakteria individual sira hamutuk 4,169, nain 2,170 (ka 52%) mai hosi pasiente mane sira no hamutuk 1,943 (47%) hosi pasiente feto sira. Enterobacterales (1146/4169, 27%) hatudu susceptibilidade ba ceftriaxone 65% (410/631) iha Escherichia coli no 25% (135/541) iha Klebsiella pneumoniae. Rezisténsia ba karbapenem hetan iha individu nain 235, inklui kompleksu Acinetobacter baumannii 61 ne'ebé rezistente ba ai-moruk oioin. Susetibilidade ba meropenem iha E. coli tun hosi 100% (33/33) iha 2020 ba 87% (155/178) iha 2024 (p=0·0002). Individu bakteria internamentu sira hosi E. coli no K. pneumoniae hatudu suseptibilidade ceftriaxone ne'ebé ki'ik liu duké bakteria individu ambulatóriu sira. Staphylococcus aureus sai nafatin susetível ba metisilina (83%, 712/858).

**Interpretasaun**

AMR fo todan ba Timor-Leste ho nível ne’ebé bo’ot, karakteriza ho rezisténsia sefalosporina jerasaun datoluk ne'ebé luan, Enterobacterales rezistente ba karbapenem ne'ebé mosu, no A. baumannii ne'ebé rezistente ba ai-moruk oioin endémiku - preokupasaun signifikativu iha ambiente ida ho opsaun tratamentu ne'ebé limitadu. Investimentu sustentavel iha prevensaun infesaun, kuidadu antimikrobiál, no sistema laboratóriu sira maka esensiál atu hamenus emerjénsia rezisténsia nian no orienta tratamentu.

**Finansiamentu**

Serbisu ida-ne'e hetan apoiu hosi Fleming Fund Country Grant (UK Aid).
